# In Vitro Tumor Models on Chip and Integrated Microphysiological Analysis Platform (MAP) for Life Sciences and High-Throughput Drug Screening

**DOI:** 10.3390/bios13020231

**Published:** 2023-02-06

**Authors:** Huyen Ngo, Sarnai Amartumur, Van Thi Ai Tran, Minh Tran, Yen N. Diep, Hansang Cho, Luke P. Lee

**Affiliations:** 1 Institute of Quantum Biophysics, Sungkyunkwan University, Suwon 16419, Republic of Korea; 2 Department of Biophysics, Sungkyunkwan University, Suwon 16419, Republic of Korea; 3 Department of Intelligent Precision Healthcare Convergence, Sungkyunkwan University, Suwon 16419, Republic of Korea; 4 Department of Medicine, Harvard Medical School, Brigham and Women’s Hospital, Boston, MA 02115, USA; 5 Department of Bioengineering, University of California at Berkeley, Berkeley, CA 94720, USA; 6 Department of Electrical Engineering and Computer Science, University of California at Berkeley, Berkeley, CA 94720, USA

**Keywords:** organs-on-chip, cancer-on-chip, breast cancer, brain cancer, tumor microenvironment, metastasis, drug development, personalized medicine, high-throughput drug screening

## Abstract

The evolution of preclinical in vitro cancer models has led to the emergence of human cancer-on-chip or microphysiological analysis platforms (MAPs). Although it has numerous advantages compared to other models, cancer-on-chip technology still faces several challenges such as the complexity of the tumor microenvironment and integrating multiple organs to be widely accepted in cancer research and therapeutics. In this review, we highlight the advancements in cancer-on-chip technology in recapitulating the vital biological features of various cancer types and their applications in life sciences and high-throughput drug screening. We present advances in reconstituting the tumor microenvironment and modeling cancer stages in breast, brain, and other types of cancer. We also discuss the relevance of MAPs in cancer modeling and precision medicine such as effect of flow on cancer growth and the short culture period compared to clinics. The advanced MAPs provide high-throughput platforms with integrated biosensors to monitor real-time cellular responses applied in drug development. We envision that the integrated cancer MAPs has a promising future with regard to cancer research, including cancer biology, drug discovery, and personalized medicine.

## 1. Introduction

### 1.1. In Vitro Models in Cancer Research

Cancer is a leading cause of death worldwide. Despite the growing understanding of cancer biology, metastasis and drug resistance remain inadequately addressed by animal or traditional cell culture studies. Although animal models have been widely used in cancer drug development, the low animal-to-human transitional rates from preclinical to clinical treatment have led to increasing concerns regarding the use of animals as predictive tools for human responses. Animals are distinct from humans in terms of genetics, epigenetics, and physiology. Moreover, the hypothesis that animal findings can be translated to humans has not yet been validated [1,2]. These issues raise the need for physiologically relevant human in vitro models to investigate cancer biology and therapeutic development. Monolayer culture assays are widely used in cancer research and drug development. However, this model poorly recapitulates the tissue structure, architecture, topography, and stiffness of its in vivo counterparts. In addition, static culture induces selective pressure on cells, affecting cell heterogeneity and resulting in modified gene and protein expression. Consequently, the development of an in vitro cancer model that precisely replicates human cancer is essential.

Recently, substantial effort has been made to develop in vitro cancer models to mimic cancer microenvironments, including two-dimensional (2D) Transwell-based platforms, three-dimensional (3D) organoid-based models, hybrid platforms, and microfluidics-based systems [3]. Transwell culture refers to a two-compartment monolayer cell culture paradigm separated by a pore membrane. It allows the co/tri-culture of cancer cells and other cells, such as stromal cells, to visualize their interactions [4]. They have also been widely used for modeling cancer cell migration and invasion [5]. Cancer cells from the upper chamber can migrate through the extracellular matrix (ECM)-coated porous membrane into the lower chamber toward chemo-attractant gradients or the endothelium, mimicking the invasion of native tumors [6]. Although it possesses some advantages compared to a single-chamber 2D culture, Transwell culture has a static culture condition that lacks nutrient exchange and stresses cultured cells. To address the limitations of monolayer tumor culture, 3D tumor models have emerged that closely mimic the tumor microenvironment (TME) by involving cell–cell and cell–matrix interactions in a spatially relevant manner [7]. Spheroids are an early but well-characterized cancer model and are still widely applied in cancer research and drug development, owing to their simplicity and structural similarity to their in vivo counterparts [8,9]. Tumor spheroids can be constructed from cancer cells alone or in combination with stromal cells with or without a scaffold [10,11,12]. The critical feature of tumor spheroids that determines their cellular behaviors, as well as drug responses, is the biochemical gradient (oxygen, nutrients, drug, and metabolites) generated along the spheroids’ depth when they grow large enough. It leads to the formation of distinct areas of cells (outer layer comprised of rapidly dividing cells, intermediate layer, and necrotic core). Hypoxia and necrosis are strongly correlated with chemotherapy resistance.

Although tumor spheroids are widely used in cancer research because of their ability to recapitulate cell–cell and cell–ECM interactions of the native brain, they have some limitations owing to their intrinsic properties: lack of tissue–tissue interface and lack of vessels, which play a central role in cancer development and invasion. Organoids are 3D self-organizing structures that originate from stem cells in vitro [13,14,15]. They are a collection of differentiated cell types that recapitulate the overall architecture and tissue-specific function of their in vivo counterparts. Cancer organoids can be expanded from patient-derived tumors or by applying CRISPR-based gene modification technology [16,17] to healthy cells to generate engineered organoid cancer models. Despite overcoming the major disadvantages of 2D monolayer and 3D spheroid cancer models in terms of the overall structure and tissue–tissue crosstalk, organoids still lack the interface between cancer cells and surrounding vessels, which is at the center of the cancer invasion and metastasis cascade. In addition, organoids are grown under static conditions without a continuous exchange of nutrients and waste, limiting cancer cell outgrowth and invasion. 

### 1.2. The Emergence of Organ-on-Chips

The evolution of cell culture models has led to the emergence of “organ-on-chips” (OOCs) or “tissue chips” to address the challenges in animal and conventional cell culture models. In 1996, a cell culture analog system was introduced to study the toxicological responses in animals and humans [18,19]. In this device, cells were cultured in an organ compartment, while cell culture media circulated through the compartments and served as a “blood surrogate”. In 2005, a high-throughput microfluidic cell culture array was used for human carcinoma HeLa cell culture, which could potentially assay 100 different cell-based experiments simultaneously [20]. The first human microfluidic lymph node was identified in 2006 [21]. This lymph node device is a membrane-based perfusion culture system consisting of a matrix-assisted central culture space (CCS) and outer culture space (OCS). Matrix-embedded dendritic cells (DCs) were mounted in the CCSs, whereas media and suspended cells were used in the OCS. The bioreactor was operated for over 14 days, and it was found that DCs clustered around the matrix fibers while T-cells and B-cells swarmed within the DC network. In the following years, the first lung (2007) [22], intestine (2008) [23], bone (2008) [24], and smooth muscle-on-chip (2009) [25] were introduced, providing the foundation for the development of organ-on-chip technology. Since the introduction of the human lung on-chip in 2010, OOCs have exponentially developed and expanded in various fields, ranging from cell biology research to drug discovery and personalized medicine [26]. The application of OOCs in cancer research (cancer-on-chips or COCs), which aims to emulate the key anatomical and pathophysiological features of human cancer, has provided insights into cancer biology while more accurately predicting the toxicity and efficacy of anticancer drugs [27,28]. 

OOCs or microphysiological analysis platforms (MAP) are compartmentalized microfluidic devices containing perfused microchannels populated by differentiated cells/stem cells [29,30,31,32,33,34] and integrated biosensors to monitor real-time physiological and metabolic activities along with molecular signaling. They consist of three main characteristics: spatial separation of different tissues, tissue–tissue interface within an organ achieved by precise geometrical control, fluid flow across the tissues mimicking their in vivo counterpart, and integrated biological and biophysical (electrical, mechanical) factors. OOCs do not contain a whole organ but only recapitulate a functional unit that matches the biological or pharmaceutical demands (such as the lung alveoli, kidney glomerulus, intestinal mucosa, and neurovascular unit) [26,35,36,37,38]. Multiorgan-on-chips can be created by connecting different single tissue chips with microchannels [34]. This process is aimed at studying the systemic crosstalk between the organs of interest and analyzing pharmacodynamics.

## 2. Cancer-on-Chips: Addressing Biological Features of Various Types of Cancer

### 2.1. Key Biological Hallmarks of Various Types of Cancer

Although cancer is a life-threatening disease, its severity is heavily dependent on the type of cancer, or in other words, the primary organ from which cancer cells emerge. Cancer of different organs is distinct in terms of incidence rate, clinical manifestations, metastasis potential, adaptation to treatment, recurrence, and prognosis. For example, malignant tumors occur at a relatively high frequency in the breast, colon, stomach, lung, and liver, but are rarely found in the heart and small intestine. Lung and liver cancers are highly metastatic, usually diagnosed on the development of symptoms induced by tumors in the metastatic organs, and have a low 5-year survival rate, while thyroid cancer has a much higher survival rate and prognosis. These differences are caused by both intrinsic factors within the organs and extrinsic factors involving crosstalk between organs in the human ecosystem. Except for the brain, most cancers of other organs (80%) are derived from epithelial tissues, which are termed carcinomas. First, cancer cells arise and grow within the epithelium, which is separated from the stromal blood vessels by the basement membrane; this is considered stage 0. During development, the cancer cells breach the basement membrane and migrate through the stroma. Once the malignant cells reach the blood/lymphatic vessels, they intravasate and are circulated throughout the human body; cells that survive the shear stress and attack of immune cells finally extravasate to tissues of distant organs. In the host organs, via communication with the surrounding parenchyma cells and stroma factors, cancer cells might survive in a foreign environment where they reinitiate their proliferative program. Microscopic metastases occur before becoming macroscopic, clinically detectable neoplastic growths. Cancer cells from primary organs are mainly disseminated via the hematogenous route, in which malignant cells are transported through the blood and lymphatic vessels to the organ site. In abdominal organs, especially the ovaries, transcoelomic metastasis may occur, in which the neoplastic cells invade the peritoneal cavity and largely reside in the peritoneal tissue and omentum [39]. Unlike most cancers, brain cancer, particularly glioblastoma (GBM), the most common malignant tumor in the adult brain, though highly invasive, rarely metastasizes outside the brain region. This pathogenic hallmark distinguishes brain cancer from malignant tumors derived from other organs and is also a fundamental characteristic for building malignant tumors on microfluidic devices. While most cancer chips focus on recapitulating the invasive-metastasis cascade by modeling the TME in primary and distant organs, brain cancer chips or GBM chips concentrate on building cancer niches located within the tumor in communication with blood vessels for cancer cell invasion within the brain. 

### 2.2. Breast Cancer-on-Chip: Modeling Disease Stages

Most breast cancers arise from epithelial cells of the milk ducts and lobules and are known as ductal carcinomas or lobular carcinomas [40,41,42]. The breast cancer-on-chip attempts to replicate different stages of breast cancer, including carcinoma in situ, invasive cancer, and distant metastasis. To recapitulate the invasion-metastasis cascade, breast cancer chips try to create a TME at the primary cancer site and distant organs, in which cancer cells communicate with other cell types with biochemical and biophysical factors for growth and local and distant invasion. 

#### 2.2.1. Breast Cancer Chips Modeling Stroma Invasion

**Figure 1:**
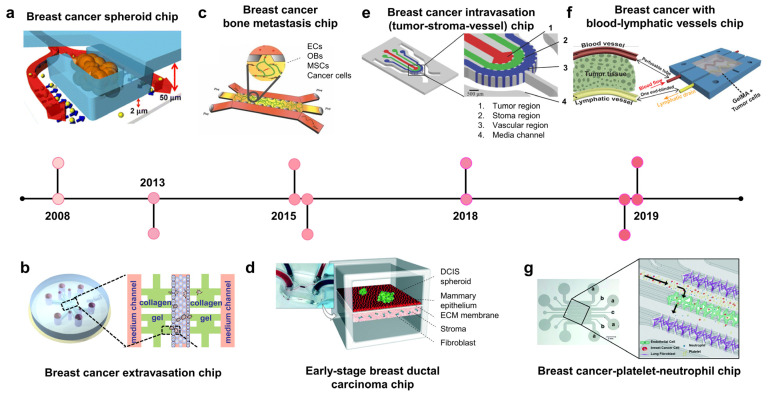
Breast cancer-on-chip. A timeline showing the development of breast cancer on chips. (**a**) Breast cancer spheroid chip: a platform consists of a U-shaped array for tumor spheroid culture and perfused system mimicking blood vessels for media and drug supply (adapted with permission from Ref. [43]. Copyright 2007, Springer Nature). (**b**) Breast cancer extravasation chip: the device comprises a central channel lined with endothelial cells and four microchambers containing an extracellular matrix (ECM). MDA-MB-231 breast cancer cells were introduced into this channel, and they transmigrated into the ECM chambers across the endothelium (adapted with permission from Ref. [50]. Copyright 2013, CC-BY-4.0). (**c**) Breast cancer-bone metastasis chip: bone-on-chip modeling the extravasation and micrometastasis of MDA-MB-231 breast cancer cells in bone tissue (adapted with permission from Ref. [56]. Copyright 2015, National Academy of Sciences). (**d**) Early-stage breast ductal carcinoma chip (DCIS): the device consists of upper and lower microchannels populated with mammary epithelial cells and stroma-embedded mammary fibroblasts, respectively; patient-derived DCIS spheroid was introduced into the epithelial layer. The cancer cells remained within the spheroid without invading the surrounding epithelium (adapted with permission from Ref. [44]. Copyright 2015, Royal Society of Chemistry). (**e**) Breast cancer intravasation chip: a microfluidic device consisting of three distinct compartments for studying tumor–stroma–vessel crosstalk. Breast cancer cells migrate from the inner tumor region into the stroma region containing ECM and invade the blood vessel channel lined with human umbilical vein endothelial cells (adapted with permission from Ref. [60]. Copyright 2018, Wiley). (**f**) Breast cancer with blood–lymphatic vessel chip: this device consisted of a pair of bioprinted blood and lymphatic vessels embedded in matrix containing MCF-7 breast cancer cells, which was also used to evaluate the role of lymphatic vessels in anticancer drug treatment (adapted with permission from Ref. [63]. Copyright 2019, Wiley). (**g**) Breast cancer–early metastasis chip (with platelets, neutrophils): this device models the early metastasis event from which platelets activate the extravasation process of cancer cells (adapted with permission from Ref. [69]. Copyright 2021, Royal Society of Chemistry).

To model stroma invasion, breast cancer chips try to reconstitute the stroma components within the TME, including stroma cells (e.g, fibroblast), ECM, growth factors, and interstitial flow (Figure 1a, 2008; Figure 1d, 2015) [43,44]. In 2008, Wu et al. developed the microfluidic self-assembly of spheroids. Cancer cells were confined in U-shaped compartments by hydrodynamic force, and continuous perfusion from a device mimicking blood vessels facilitated spheroid formation (Figure 1a, 2008) [43]. This method effectively produced many homogenous spheroids for preclinical anticancer drug screening. The most remarkable group developed a breast TME with three components: blood vessels, stroma, and tumor region [45] BT549 and T47D breast cancer spheroids were formed in U-shaped chambers within the tumor region, which absorbed nutrients and drugs from blood vessels. Nanoparticle-carrying doxorubicin injected into blood vessels could penetrate the ECM region to reach the tumor region and induce cytotoxicity in cancer cells but not in healthy endothelial cells.

Ductal carcinoma in situ (DCIS) is a preinvasive lesion that can potentially become invasive. In DCIS, the malignant cells reside within the epithelial layer and do not cross the basement membrane. Once the basement membrane is destroyed, cancer cells invade the ECM and migrate toward the blood/lymphatic vessels. A breast cancer–stroma chip was successfully remodeled for DCIS in vitro, in which breast cancer spheroids continued to grow and remained within the mammary epithelial layer without crossing the basement membrane during culturing (Figure 1d, 2015) [44]. The device consisted of two parallel microchambers separated by a thin polydimethylsiloxane (PDMS) membrane populated with mammary epithelial cells and stroma-embedded mammary fibroblasts. Patient-derived DCIS spheroids were introduced into the epithelial layer, where cancer cells remained within the spheroid without invading the surrounding epithelial layer. These studies show that breast tumor–stroma chips may be used to investigate the crosstalk between breast cancer cells and stromal components, such as ECM, fibroblasts, growth factors, and interstitial fluid.

#### 2.2.2. Vascularized Breast Cancer Chips Modeling Metastasis 

Extravasation is a critical process for cancer metastasis [46,47,48]. Extravasation usually occurs in capillaries, where the vessel diameters are similar to the cell size and cancer cells slow down and attach to endothelial cells. Cancer cells may move along with endothelial cells to find the optimal site for extravasation before disrupting endothelial junctions by invadopodia and migrating from the vessel lumen into the tissue of distant organs [47]. A breast cancer–blood vessel system was used to model the adhesion of circulating cancer cells to the endothelium via CXCL12–CXCR4 interaction [49]. A model with endothelial-lined and ECM-filled channels has shown that MDA-MB-231 cells increased the permeability of the vessels, allowing for their transmigration into the stroma (Figure 1b, 2013) [50]. In another microfluidic device, a self-assembled vascular network of human umbilical vein endothelial cells (HUVECs) was formed, allowing for spatiotemporal monitoring of breast cancer cell extravasation under hypoxia, which usually occurs in the TME [51]. Hypoxia promotes cancer cell invasion, EMT, and extravasation by upregulating hypoxia-inducible factors (HIFs). 

Once breast cancer cells invade the bloodstream, they circulate throughout the body before extravasating at the capillaries of distant organs. According to the “seed-soil” theory, metastasis does not occur randomly. Organ-specific factors can determine the preferential sites of a type of cancer [52,53,54,55]. A bone-on-chip using osteo-differentiated human-bone-marrow-derived mesenchymal stem cells with endothelial cells in vessel-mimicking channels showed a higher extravasation rate of MDA-MB-231 cells than that of a collagen-only matrix [50]. This study also confirmed that osteoblast-derived CXCL5 enhances the extravasation of breast cancer cells via its CXCR2 receptor on cancer cell membranes. In another study, both bone and skeletal muscle microenvironments were created to compare the effects of different “soils” on MDA-MB-231 “seeds” (Figure 1c, 2015) [56]. As expected, this model showed that the extravasation rate of breast cancer cells in an osteoblast-conditioned matrix was significantly higher than that in a myoblast-conditioned matrix. Using this model, they found that the antimetastatic effect of skeletal muscles was related to A3AR expressed on MDA-MB-231 cells.

Intravasation is a critical step in distant metastasis, in which cancer cells transmigrate through the basement membrane of the blood and lymphatic vessels into the circulation [57,58,59]. Cancer cells move toward new blood vessels, attach to endothelial cells, and disrupt endothelial junctions. A breast tumor–stroma–vessel chip consisting of three distinct compartments was used to model intravasation (Figure 1e, 2018) [60]. A vessel compartment was introduced outside the stroma surrounding the tumor core. MDA-MB-231 cells originated from the tumor region, migrated through the stroma region, and intravasated. The endothelial cells also promote the invasion of cancer cells into the stroma. The entire intravasation process was visualized on a breast cancer chip, in which blood vessels were embedded within collagen type I [61]. MDA-MB-231 cells degraded the local ECM, creating narrow tunnels that allowed them to move back and forth. When a cancer cell reaches the endothelium, it slows down and moves along the ECM/vessel interface, replaces the proximal endothelial cells, and protrudes its body into the vessels.

Blood vessels bring nutrients, oxygen, and drugs into the TME, whereas lymphatic vessels drain interstitial fluid from the tissues. To improve the existing in vitro cancer drug screening platform, breast tumor chips with blood and lymphatic vessels were designed to better recapitulate the in vivo drug transport and absorption [62,63]. A two-layer tumor chip consisting of both blood and lymphatic vessels was constructed to test the response and resistance of breast cancer cell lines to doxorubicin [62]. The survival fraction of cancer cells grown on this chip was higher than that of cells grown in conventional 2D models. In another study, a pair of bioprinted blood and lymphatic vessels were embedded in matrix-containing MCF-7 breast cancer cells (Figure 1f, 2019) [63]. This platform was also used to evaluate the anticancer effect of doxorubicin. It was found that lymphatic vessels increased cancer cell viability, possibly because of the drainage of the drug, resulting in a reduced drug concentration at the tumor site.

In addition to host tissues, blood cells, including platelets and immune cells (macrophages and neutrophils), are increasingly recognized to play regulatory roles in cancer progression, metastasis, and drug resistance [64,65]. These cells show the activation or inhibition of cancer cell extravasation. Some studies have reported the enhancement of platelets in cancer metastasis in xenograft tumor models [66]. However, whether neutrophils promote or inhibit cancer metastasis remains unclear [67,68]. In this scenario, Crippa et al. designed a platform modeling early metastasis events at the blood vessel–tissue border, in which circulating breast cancer cells escape from the vessel to invade the tissue (Figure 1g, 2019) [69]. The endothelium was seeded into the central chamber to form a self-assembled vascular network through which the cancer cells, platelets, and neutrophils flowed. Interestingly, this platform demonstrated the role of platelets in enhancing breast cancer metastasis. This study also demonstrated the effect of an antiplatelet drug, eptifipatid, on cancer cells and blood vessels. It suppressed the expression of cancer-invasive genes while promoting the tight junctions of endothelium. 

### 2.3. Brain Cancer-on-Chip: Recapitulate Glioblastoma Niches

GBM is the most common and aggressive type of adult brain cancer [70]. Unlike other solid tumors, complete surgical resection of GBM is impossible because of the invasion and infiltration capabilities of this type of tumor [70]. In addition, GBM cells are highly resistant to radiotherapy and chemotherapy. The glioma microenvironment comprises distinct regions called tumor niches. GBM contains GSCs, which contribute to tumor development and therapeutic resistance. They exist in at least three distinct niches classified based on vasculature features: perivascular, invasive, and hypoxic [71,72]. In the perivascular niche, GSCs are in close contact with abnormal hyperplastic microvessels resulting from hyperangiogenesis [73]. In the invasive niche, GSCs are located in normal blood vessels, using the vasculature as a highway to spread into the healthy parenchymal region [74]. The hypoxia niche consists of non-functional or thrombotic vessels that induce necrotic areas from which GSCs migrate, forming pseudopalisading [75,76]. Brain cancer chips try to recapitulate three types of GSCs niches, in which GSCs are in close contact with the artificial vasculature. These systems have successfully modeled the microenvironment and the pathological and histological hallmarks of GBM.

**Figure 2:**
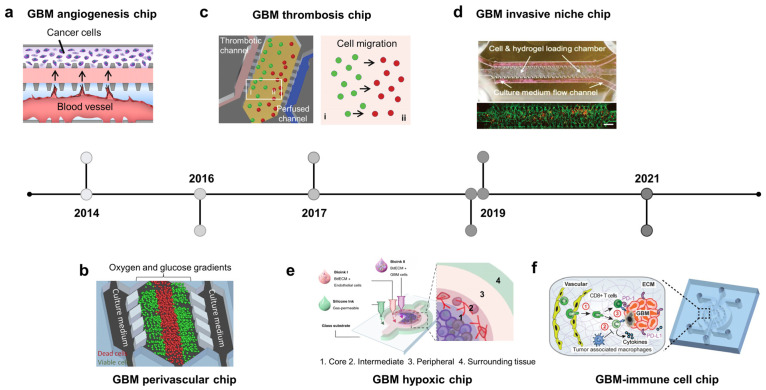
Brain cancer-on-chip. A timeline showing the hallmarks of glioblastoma (GBM) on the chips. (**a**) GBM angiogenesis chip: microvessels were formed adjacent to tumor cells, allowing f the generation of the soluble factors’ gradient between two channels (adapted with permission from Ref. [77]. Copyright 2014, AIP Publishing). (**b**) GBM perivascular chip: gradients of oxygen and glucose were generated on a microfluidic chip. Dead cells were observed in the central chamber with low concentrations of nutrients/oxygen (adapted with permission from Ref. [79]. Copyright 2016, CC BY 4.0). (**c**) GBM thrombosis chip: thrombotic channels were fabricated by collagen hydrogel and a controlled medium flow, mimicking blood-vessel obstruction events. Starvation of oxygen and nutrients triggered the cell migration towards the perfused channel (adapted with permission from Ref. [83]. Copyright 2017, Oxford University Press). (**d**) GBM invasive niche chip: endothelial cells and brain tumor cells were loaded in the cells/gel chamber, connecting to the media flow channel by triangular micro-posts. Brain tumor cells are preferentially localized in the perivascular zone where the blood vessels also serve as routes for cancer cells to migrate to other brain regions (adapted with permission from Ref. [81]. Copyright 2019, CC BY 4.0). (**e**) GBM hypoxic chip: cancer and endothelial cells were printed with the brain-derived extracellular matrix, mimicking the hypoxic necrosis and the microvascular hyperplasia in the tumor microenvironment (TME) (adapted with permission from Ref. [84]. Copyright 2019, Springer Nature). (**f**) GBM–immune cell chip: brain tumor microvessels were fabricated on a chip to explore the crosstalk of CD8^+^ T cells and the GBM TME (adapted with permission from Ref. [85]. Copyright 2020, CC BY 4.0).

The GBM niche is where GSCs are in direct contact with the endothelial cells. The perivascular niche is characterized by hyperangiogenesis, which has been recapitulated on a microfluidic device (Figure 2a, 2013) [77]. In this device, GBM and endothelial cells were cultured in two distinct channels separated by the ECM. The introduction of U87 cancer cells promoted angiogenic sprouts from the endothelium, which may be attributable to the actions of vascular endothelial growth factor (VEGF). Furthermore, GSCs co-cultured with endothelial cells in perivascular niche environments can maintain stemness and enhance migration and chemotherapy resistance [78]. Another perivascular niche chip recapitulated the oxygen and nutrient gradients of GBM tumors (Figure 2b, 2016) [79]. Cancer cells were embedded in collagen and grown in the central chamber, while media were perfused through two lateral chambers and diffused into the tumor region. This study showed a reduction in the viability of cells distant to the oxygen and nutrient supply. Invasive niche-on-a-chip revealed that blood vessels not only increased the expression of neural stem cell markers but also promoted invasion by GSCs, which was related to the CXCL12-CXCR4 signaling pathway [80]. A GBM model also captured the colocalization between GSCs and blood vessels, with vessels directly serving as a path for migrating tumor cells into the normal brain parenchyma (Figure 2d, 2019) [81]. This study revealed signatures related to the “homing” of GSCs to blood vessels, including proneural/mesenchymal tumor cells and the platelet-derived growth factor receptor alpha PDGFRS gene. Two specific histological hallmarks that distinguish GBM from lower-grade gliomas are microvascular proliferation and pseudopalisading necrosis. Therefore, in addition to the perivascular niche, several efforts have been made to recapitulate the GBM hypoxic niche-on-chip to study cell behavior in the necrotic region and uncover the underlying mechanisms of chemotherapeutic resistance. For example, a GBM hypoxic niche on a chip generated by gravity-driven perfusion of culture media through GSC spheroids showed an increase in cancer stem cell markers (Nestin and CD133), proinflammatory cytokines IL6, and hypoxia-induced factor 1-alpha (HIF-α). This hypoxic microenvironment induces HIF-α- and IL6-dependent resistance to doxorubicin [82]. Pseudopalisading necrosis is characterized by the alignment of glioma cells with elongated nuclei-like palisades in neat rows around the necrotic center. Some mechanisms for this phenomenon have been proposed, including edema-enhanced vessel collapse, vasoocclusion, vascular regression, and intravascular thrombosis, with more than 50% of pseudopalisades in histological samples from GBM patients with thrombosed vessels in necrotic areas. A GBM microfluidic chip was established to mimic the vessel occlusion occurring within the GBM microenvironment, allowing for the monitoring of glioma cell behavior during thrombosis (Figure 2c, 2017). This device consists of a central microchamber with GBM cells and two lateral channels perfused with culture medium to mimic the brain blood vessels. Twenty-four hours after seeding the cells, two inlets of a lateral channel were sealed, enabling medium perfusion through the other channel to mimic the thrombosis event. This triggered a migration wave of glioma cells located near the occlusion channel towards the perfused channel, showing the formation of a pseudopalisade front in vitro [83]. Another GBM hypoxic niche chip was created using 3D bioprinting technology (Figure 2e, 2019) [84]. The tissues were printed onto a non-permeable glass substrate into two distinct compartments: the tumor inside was surrounded by vascular endothelial cells and enclosed by an outermost silicone-wall chamber filled with culture medium. The entire structure was covered by a non-permeable glass substrate so that nutrients and oxygen could only be absorbed into the tissue via the gas-permeable silicone wall, which generated an oxygen gradient inside the tumor. The GBM forms anatomically distinct regions (core, intermediate, and peripheral) along the oxygen gradient. Pseudopalisading necrosis was observed in the core region. Simultaneously, the invasion and hyperplasia of microvessels were observed in the peripheral region due to the excessive proliferation of cancer cells in the intermediate region. The development of GBM chips has led to the introduction of the immune system into the TME. Recent studies have involved immune and adaptive immune system components, in addition to GBM cells and the vasculature, to study the crosstalk between them, focusing on their immunosuppressive role in cancer growth and therapeutic development (Figure 2f, 2021) [85]. The device consists of a central compartment populated by patient-derived GBM cells, two lateral compartments with human brain endothelial cells, and two outermost compartments housed by tumor-associated macrophages. This study focused on the effect of the genetic background on immunomodulation and showed that molecularly distinct GBM subtypes have distinct epigenetic and immune signatures that may lead to different immunosuppressive mechanisms.

### 2.4. Other Types of Cancer on Chips: Modeling Invasive-Metastasis Cascade

#### 2.4.1. Ovarian Cancer Chip 

**Figure 3:**
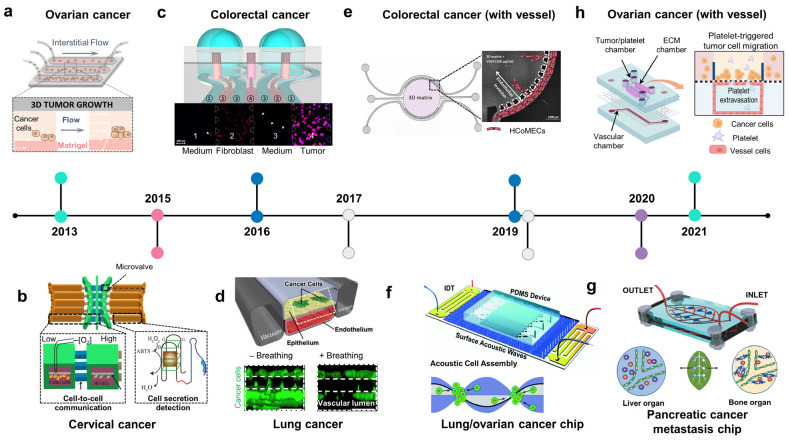
The development of organ-on-chip for studies of lung, digestive, and gynecologic cancer. (**a**) Ovarian cancer chip: the intestinal flow was applied to form a 3D ovarian tumor spheroid in a Matrigel-laden microchannel (adapted with permission from Ref. [89]. Copyright 2013, National Academy of Sciences). (**b**) Cervical cancer chip: cervical cancer on chip recreated low oxygen conditions to study the cell–cell interaction of cervical cancer cells and endothelial cells under hypoxia and detect secreted molecules from the cells (adapted with permission from Ref. [92]. Copyright 2015, CC BY 4.0). (**c**) Colorectal cancer chip: co-culture of human fibroblast and colorectal cancer showed the mobility of normal fibroblasts toward tumor cells (adapted with permission from Ref. [95]. Copyright 2016, CC BY 4.0). (**d**) Lung cancer chip: mechanical breathing inhibited the proliferation of lung cancer cells in an alveolar/bronchiolar chip (adapted with permission from Ref. [100]. Copyright 2017, Elsevier). (**e**) Colorectal cancer chip with a vessel: VEGF-produced colorectal tumor-inducing invasion of endothelial cells in a circular chip (adapted with permission from Ref. [97]. Copyright 2019, CC BY-NC 4.0). (**f**) Acoustofluidic ovarian/lung cancer chip: an acoustofluidics-based cancer platform allows for the formation of high-throughput 3D tumor spheroids and a large-scale in vitro cancer model analysis (adapted with permission from Ref. [112]. Copyright 2019, Royal Society of Chemistry). (**g**) Pancreatic cancer chip: a leaf-inspired symmetrical microfluidic platform mimicked vascularization of the liver and bone and extravasation of pancreatic cancer cells (adapted with permission from Ref. [108]. Copyright 2020, Wiley). (**h**) Ovarian cancer chip with a vessel: the platelet-triggering ovarian migration was demonstrated in a multicellular microfluidic platform (adapted with permission from Ref. [90]. Copyright 2021, CC BY 4.0).

Ovarian and cervical cancers are the most common and lethal gynecological cancers [86,87]. Epithelial ovarian cancer accounts for approximately 95% of ovarian cancers, of which serous adenocarcinoma is the most common histological type [88]. Ovarian cancer-on-chip has modeled cancer progression and metastasis via crosstalk with both peritoneal fluid (transcoelomic route) and blood components (hematogenous route) (Figure 3a, 2013; Figure 3h, 2021). A 3D ovarian cancer platform was used to culture cells under laminar flow and study the role of interstitial fluid in modulating cancer metastasis. The interstitial fluid restricted tumor volume and viability while transforming cancer cells into more aggressive phenotypes [89]. The role of platelets in cancer development and metastasis is being increasingly recognized. Therefore, a recent ovarian cancer chip was designed to study the metastasis of ovarian cancer via the hematogenous route, focusing on the role of platelets (Figure 3h) [90]. This study revealed that ovarian cancer cells overexpressed galectin 3, which binds to the collagen receptor glycoprotein VI (GPVI) on platelets, enhancing metastasis. Pharmaceutical inhibition of GPVI arrests metastasis and supports chemotherapy. Accumulating evidence suggests that hypoxia in the TME is strongly related to poor prognosis in patients with ovarian cancer. An ovarian tumor-on-chip was used to mimic the oxygen gradient in vivo by rolling a biocomposite strip onto an oxygen-impermeable metallic core and submerging the engineered tumor into a culture medium [91]. The biocomposite strip was generated by infiltrating a cancer cell–collagen gel suspension into a thin porous cellulose scaffold strip. After culturing for the desired time, tumor rolls were disassembled for analysis. This study revealed that cells in the outer layers underwent mild hypoxia, whereas those from the deep layers were severely hypoxic at 6 and 12 h and adapted to hypoxic conditions at 24 h. Metabolic adaptation to oxygen availability is mediated by the well-established transcription factors HIF and unfolded protein response target genes UPR. A cervical cancer model was designed to study the migration of CaSki cervical cancer and endothelial cells under hypoxic conditions (Figure 3b, 2015) [92]. Tumor and endothelial cells were cultured in separate chambers connected by narrow channels of different lengths. Under the 5% oxygen condition, CaSki cells moved faster than HUVECs; meanwhile, under the 15% oxygen condition, HUVECs migrated faster. HIF-1alpha, VEGF-165, and reactive oxygen species (ROS) were analyzed to elucidate gene regulation under hypoxia, which affected the migration of CaSki and HUVEC. 

#### 2.4.2. Gastrointestinal Cancer Chips 

Malignant tumors of the digestive system usually occur in the esophagus, stomach, large intestine, pancreas, and liver. Cancer of the small intestine is relatively rare compared to that of other organs. Esophageal cancer has two main types: adenocarcinoma and squamous cell carcinoma. It usually metastasizes to the lungs, liver, bones, adrenal glands, and brain. The most common type of gastric cancer is adenocarcinoma, which primarily spreads to the liver [93]. Metastatic colorectal cancer most commonly occurs in the lungs, bones, brain, or spinal cord Organ chips applied to gastrointestinal cancer focus on investigating tumor cell–stroma cell crosstalk-inducing EMT, which is involved in the invasion-metastasis cascade and therapeutic resistance. The gastric cancer-ECM droplet model showed that ECM promoted EMT of AGS and Hs746T gastric cancer cells, which also increased their resistance to 5-FU [94]. Colorectal cancer has also been modeled on microfluidic chips involving tumor–stroma and tumor–vessel crosstalk (Figure 3c, 2016; Figure 3e, 2019). A co-culture system with normal human fibroblasts and HT-29 colorectal cancer cells showed cell–cell interactions between them and that fibroblasts acquired a cancer-associated activation state (increased alpha-SMA expression and motility) and HT-29 cells enhanced their proliferation and resistance to paclitaxel (Figure 3c, 2016) [95]. The progression and invasion of cancer results from the interaction between cancer cells and other tissue factors, including biochemical and biophysical barriers. Besides biochemical factors secreted by cancer and stromal cells, biophysical factors, including stromal stiffness, interstitial flow, and fluids, also affect the growth and development of cancer. Esophageal cancer cells growing under a laminar flow showed higher expression levels of mesenchymal and stem cell markers and more resistance to docetaxel than cancer cells growing under static conditions [96]. VEGF produced by tumors is a critical factor that regulates cancer angiogenesis. Co-culturing HCT-116 colorectal cancer cells with human colonic microvascular endothelial cells on a circular chip revealed the invasion of endothelial cells toward HCT-116-secreting VEGF cells (Figure 3e, 2019) [97]. Gemcitabine (GEM)-coated nanoparticles applied to the chip showed stepwise decay in toxicity, which was less linear than that of GEM perfused directly. 

#### 2.4.3. Lung Cancer Chips 

Lung cancer is one of the deadliest cancer types, of which adenocarcinoma is the most common and accounts for approximately 40% of lung cancers [98,99]. The lung-on-a-chip is an integrated system that recapitulates alveoli, the structural and functional unit of the lung, which includes the air–liquid interface with alveolar epithelial cells and endothelial cells growing on two sides of an ECM-coated PDMS porous membrane [26]. A mechanical strain was applied laterally to the culture chambers to mimic a respiratory rhythm. Patient-derived non-small-cell lung cancer (NSCLC) cells were introduced into the alveolar epithelial layer to create orthotopic NSCLC on the alveolar/bronchiolar chip (Figure 3d, 2017) [100]. H1965 human NSCLC cells proliferated more rapidly on the alveolus chip than on the airway chip. Mechanical breathing restricted the growth of H1965 cells by downregulating epidermal growth factor receptor (EGFR) expression and promoted H1965 resistance to the third-generation tyrosine kinase inhibitor rociletinib. The metastatic potential of lung cancer in distant organs was also examined using microfluidic devices consisting of a lung chamber connected to the brain, liver, and bone chamber [101,102]. In these models, A549 NSCLC cells proliferated and crossed the ECM-coated porous membrane into the lower endothelial layer and invaded other organs with support from resident stromal cells in the host tissues. Notably, the lung-BBB-brain parenchyma model allowed for real-time monitoring of brain metastasis and revealed the role of aldo-keto reductase family 1 B10 (AKR1B10) in promoting cancer cells exiting the BBB. Another tumor–stroma lung chip revealed the role of CAFs in increasing glucose-regulated protein 78 (GRP-78) expression in A549 and SPCA-1 NSCLC cells, which facilitated their invasion [103].

#### 2.4.4. Liver and Pancreatic Cancer Chips 

The liver and pancreas are accessory organs for digestion. On-chip liver cancer attempts to create an artificial TME to study tumor–stroma cells, tumor–ECM, and tumor–vasculature association. Hepa1-6 liver cancer cells convert JS-1 hepatic stellate cells, pericytes found in the perisinusoidal space of the liver, into cancer-associated cancer stellate cells in a co-culture system [104]. In another platform, the decellularized liver matrix showed enhanced capability to maintain cell viability and function under flow. A cellulose/collagen artificial blood vessel implanted collagen I system was utilized to model the transendothelial migration of HCCLM9 liver cancer cells and invasion of endothelial cells through vascular walls toward the source of VEGF [105]. Similarly, pancreatic cancer models also recapitulate tumor–vasculature and tumor–stroma cell models, in which PANC-1 pancreatic spheroids and pancreatic stellate cells (PSC) interact with each other to mediate tumor progression [106]. Besides being aggressive and lethal, pancreatic ductal adenocarcinoma (PDAC) is known for its hypervascularity. PD7591 pancreatic cancer cells and endothelial cells were seeded in two parallel cylindrical channels embedded in the collagen matrix to mimic the pancreatic duct and blood vessels [107]. Both cell types form a monolayer inside the channels, where PD7591 cells migrate toward the blood-vessel track upon stimulation with FBS. Once they reach the vascular channel, PDAC cells pass through the vessel wall and induce apoptosis in proximal endothelial cells. This model also revealed the contribution of the TGF-β/activin/ALK7 pathway in mediating endothelial apoptosis. Another pancreatic cancer chip model was inspired by leaf venation (Figure 3g, 2020) [108]. Mimicking the leaf venation architecture, this device consists of two symmetrical channel networks along the main channel, each connected to three independent cell culture chambers. Endothelial cells were seeded into the main channel and perfused throughout the networks to form a self-assembled vasculature network mimicking the vascular system in vivo. Interestingly, the symmetrical design has allowed the study of organ-specific metastasis in a single device by culturing hepatic cells on one side and mesenchymal stem cells on the other to mimic a vascularized liver and bone, respectively. Pancreatic cancer cells were perfused into the main channels and transported via the vasculature-like system to distant organs (liver and bone), where they extravasated to invade these organs. The number of cancer cells extravasating into the bone was higher than that in the liver, indicating that metastasis depends on the host environment. 

#### 2.4.5. Urinary Tract Cancer Chip 

Urinary tract cancers, including those of the kidney and bladder, most frequently originate from epithelial cells and spread via the lymphatic or hematogenous route. To build an ex vivo paradigm for these tumors, tumor cells are implanted into an artificial TME, including stromal cells, ECM, blood vessels, and various biophysical and biochemical components. A bladder cancer microenvironment was created by co-culturing T24 bladder cancer cells with stromal cells, including fibroblasts, macrophages, and endothelial cells, in four indirectly connected chambers filled with Matrigel [109]. Macrophages in this TME expressed Arg-1, a marker of M2 macrophages, an immunosuppressive subset of tumor-associated macrophages. Primary human clear cell renal cell carcinoma (ccRCC) is the most common kidney cancer in adults and originates in the epithelial layer of the proximal convoluted tube [110]. A ccRCC-on-a-chip, consisting of an endothelial-lined lumen surrounded by ECM-embedded ccRCC cells, was designed to recapitulate ccRCC-induced sprouting. This study revealed that angiogenesis is mediated by ANGPTL4, PGF, and VEGFA [111]. 

## 3. Advantages of Cancer Chips and Applications in Drug Development

### 3.1. Effect of Flow on Cancer Growth

Unlike other in vitro models in which cells are grown in a static environment that stresses the cells and limits the exchange of O_2_, CO_2_, nutrients, and metabolic wastes, microfluidic cell culture models contain fluidic flow across microchannels in a dynamic system. The flow is one of the most important intrinsic factors of the OOC as it recapitulates the pivotal features of the human body: the circulation of blood and lymphatic flow to every part of the body to nourish the tissues, the interstitial flow within the tissues allowing for the exchange of molecules between the cells and extracellular space, and maintenance of cellular osmosis. Interstitial fluid flow is the movement of fluid across tissues that reside between blood and lymphatic vessels [113,114]. This flow not only provides a means of transporting biomolecules but also a source of mechanical cues that can trigger intracellular responses [113]. Although there has been no direct measurement of interstitial flow in vivo, numerical simulation studies have shown that the flow has a velocity of magnitude of 10-6 m/s, and experimental studies have also demonstrated that a flow of μm/s induces physiological responses from cells. There are some hypotheses on how interstitial flow affects cell bioactivities, mainly focusing on shear stress and solid stress induced by the flow, with the involvement of cell-membrane-related receptors, ion channels, the cell surface glycocalyx, integrins, and signaling messengers [113]. In the microfluidic device, interstitial flow is partially recapitulated with the introduction of medium flow through cell culture chambers. Several studies have demonstrated that microfluidic devices significantly improve cell viability compared to static conventional models, possibly owing to the constitutive supply of nutrients and removal of waste. In cancer models, microfluidic flow has been shown to promote cancer growth, aggressiveness, and metastasis [115,116]. These observations from in vitro models are in accordance with the role of interstitial fluid in promoting cancer progression observed in vivo [117]. In addition to modeling interstitial flow within tissues, the flow of microfluidic devices also helps integrate multiple organs into a single platform that allows communication between organs [101]. The multiorgan devices are especially useful to validate anticancer therapeutic efficiency as the direct cytotoxic effect of drugs on malignant cells and drug metabolism and kinetics are partially reflected on these platforms. 

### 3.2. Culture Period

In vitro cancer chip models offer numerous advantages for the study of cancer biology and drug development. A key benefit is that the cancer progresses in a short period (a few weeks to months) in the model as opposed to the in vivo development of malignant tumors over years. The emergence of cancer in vivo primarily results from the accumulation of cellular damage due to exposure to oncogenic agents, which leads to alterations in molecular patterns. This persistent, insidious process occurs many years before the malignant cells arise. After that, it takes several months to a year for tumor cells to extensively proliferate, invade, and metastasize to distant organs. In vitro platforms not only shorten this process, but also allow us to model each step and independently study the development of tumor cells in different stages. For example, malignant cells arise from the accumulation of damage caused by exposure to several risk factors before inducing oncogenic mutations that cause cancerous phenotypes. Cell culture models allow for the study of this two-step process separately: before the occurrence of the mutation by treatment with environmental agents potentially causing cellular damages and after acquiring cancerous phenotypes by directly applying malignant cell lines or patient-derived tumor cells onto the culture platforms to investigate the invasion-metastasis cascade. The latter stage has been developed in several organ chip models, allowing for real-time monitoring of cancer progression and identification of non-malignant factors in both primary and distant organs that contribute to cancer spread and therapeutic resistance [56,102]. For example, a multiorgan microfluidic device integrates the lung organ with the BBB to monitor the entire process of brain metastasis of lung cancer [102]. It took two days for cancer cells to cross through the endothelium in the lung and then one day to reach the BBB with their presence in the brain parenchyma. Proliferation was observed 24 h and 36 h later. In another microfluidic device that models secondary organs (bone, skeletal muscles) to study the metastatic ability of breast cancer to different sites, cancer cells were found to attach to blood vessels after 2 h, transmigrate through the vessel walls after 4 h, and become detectable in the outer vessel space after 6 h [56]. These models allow for the investigation of both subcellular signaling and cell–cell communication within the TME affecting cancer metastasis. For example, the expression of AKR1B10 by lung cancer cells contributes to brain metastasis, whereas adenosine released by skeletal muscle cells inhibits the migration of breast cancer cells into the skeletal muscle microenvironment. 

### 3.3. High Throughput Assay for Drug Development and Preclinical Studies

In addition to providing tissue-specific cues to identify targetable molecules and cellular signaling, cancer chips with controlled flow also offer high-throughput platforms to screen various drug candidates with different concentrations to evaluate multiple aspects of the tumor, including the drug penetration rate via the physical barrier (e.g., BBB), cancer cell survival, toxicity on healthy cells, proliferation rate, angiogenesis, drainage of the drug via lymphatic vessels, resistance, and recurrence rate (Table 1). Flow is an intrinsic property of cancer chips, which is helpful in designing a high-throughput drug screening platform. Geometrically controlled flow allows for the combination of multiple drugs at a range of concentrations [118]. Integrating this platform with patient-derived spheroids helps identify the optimal combination of drugs for individual patients [118,119]. Another design applicable for high-throughput drug screening is the spheroid array, in which spheroids of cancer cells are grown in a parallel array interconnected by microchannels continuously perfused with media [119]. These platforms provide several advantages as drug screening systems, including a physiological flow, dynamic supply of nutrients, waste removal, utility of small sample volumes, and incorporation of patient-derived spheroids. The most prominent feature of spheroids is that they resemble the oxygen and metabolite gradients seen in solid tumors and therefore mimic the hypoxia observed in vivo [120,121]. The hypoxic core promotes angiogenesis and cancer invasion and hampers the therapeutic response [122]. Therefore, spheroid arrays are valuable for evaluating patient responses to drugs and predicting therapeutic resistance. Another cancer platform for high-throughput drug screening is the multi-unit tissue and organ level of the TME. An example of this is the 24-unit BBB chip incorporating lung or GBM tumors, which permits for the evaluation of drug penetration via the BBB and anticancer responses [123]. Each unit consists of a two-channel device separated by an ECM-coated PDMS membrane, and the lower channel was lined with brain endothelial cells maintained by media flowing through the lower channel. Lung and GBM tumor spheroids were introduced into the upper channel to study their invasive ability through the BBB. Drugs perfused via the lower channel penetrated the BBB to reach tumor sites. This platform allows for high-throughput evaluation of cancer cell behavior, BBB penetration of drugs, anticancer efficacy, and cytotoxicity in healthy endothelium. Another application of cancer chips is their ability to model the ecology of cancer cells. Owing to the precise control of the geometry, microfluidic chips can recapitulate the heterogeneous behavior of cells located at different positions within the tumor. For example, a microfluidic device consisting of 488 hexagonal microchambers has been used to recapitulate tumor recurrence after treatment with doxorubicin [124]. A gradient of doxorubicin was generated through the device, which resulted in tumor resistance, as evidenced by the empty three-quarters of the chambers by day 5 and repopulation with resistant cells by day 7. Transcriptome sequencing also revealed three significant changes related to doxorubicin resistance: mutation in the filamin A gene, overexpression of the aldo-keto reductase enzyme, and activation of the NF-kB proinflammatory pathway. Taken together, these examples demonstrate that cancer chips offer powerful platforms for high-throughput drug screening and preclinical studies. 

### 3.4. Integration of Biosensors in Cancer-on-Chips or Cancer Microphysiological Analysis Platforms to Monitor Real-Time Molecular, Cellular, Physiological, and Metabolic Activities

**Figure 4:**
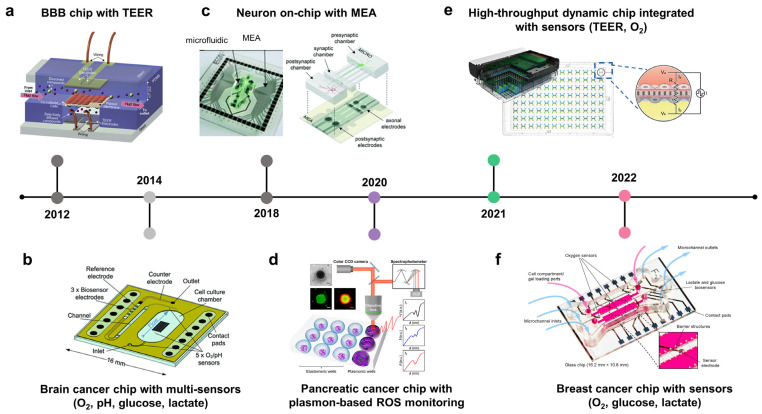
Repurposable integrated sensors on microphysiological analysis platforms (MAP) to monitor real-time physiological responses and signaling pathways. A timeline showing the development of microfluidic chips with integrated sensors for cancer modeling and drug development. (**a**) Blood–brain barrier (BBB) with transepithelial/transendothelial electrical resistance (TEER) (adapted with permission from Ref. [132]. Copyright 2012, Royal Society of Chemistry). (**b**) Cancer chip with multi-sensors (O_2_, pH, glucose, and lactate). Integrated biosensors to brain cancer on-chip or MAP for monitoring changes of pH, oxygen, glucose, and lactate induced by tumor metabolism (adapted with permission from Ref. [134]. Copyright 2014, Royal Society of Chemistry). (**c**) Neuron on-chip with multi-electrode array (MEA) (adapted with permission from Ref. [136]. Copyright 2018, Royal Society of Chemistry). (**d**) Pancreatic cancer chip with plasmon-based monitoring of reactive oxygen species (adapted with permission from Ref. [137]. Copyright 2020, American Society Chemistry). (**e**) High throughput breast cancer chip integrated with cancers (adapted with permission from Ref. [133]. Copyright 2021, CC BY-NC 3.0). (**f**) Breast cancer chip with sensors (O_2_, glucose, and lactate) (adapted with permission from Ref. [135]. Copyright 2022, CC BY 3.0).

Cancer cells release several soluble factors into their surrounding environment to facilitate their growth and invasion. These biomolecules are critical for cancer diagnosis and evaluation of disease stage and prognosis. Therefore, integrating biosensors in COC would help construct TMEs with a precise level of oxygen and nutrients while allowing for real-time monitoring of metabolites and cancer biomarkers (Table 2). Current OOC technology is integrated with transepithelial/transendothelial electrical resistance (TEER) techniques to measure the integrity of epithelium/endothelium layers (Figure 4a, 2012; Figure 4e, 2021) [130,131,132]. In addition, metabolites from cancer cells have been monitored on-chip in real-time to evaluate the progression of cancer and drug responses. For instance, cancer-on-chip platforms have been integrated with pH, oxygen, lactate, and glucose sensors to monitor the dynamics of these metabolites induced by brain/breast cancer cells (Figure 4b, 2014; Figure 4f, 2022; Figure 4g, 2022) [133,134,135]. In addition to cell metabolites, real-time measurement of cellular electrical activity within the tumor, particularly in healthy neurons, is also essential for evaluating the invasion of cancer cells into the surrounding healthy tumor and its effect on neuronal activity and brain function (Figure 4c, 2018) [136]. For therapeutic monitoring, markers of cellular stress, ROS, were also measured on-chip to evaluate pancreatic cancer cell responses to doxorubicin treatment using a plasmon-based technique (Figure 4d, 2020) [137].

#### 3.4.1. Measuring Oxygen Concentration

The high proliferative rate of cancer cells along with abnormal vessels resulting from angiogenesis leads to heterogeneous oxygen concentrations within the tumor and the existence of hypoxic cores, which contribute to cancer development, metastasis, and therapeutic resistance. Numerous studies have reported the roles of hypoxia and HIFs in tumor cell migration, angiogenesis, immune suppression, premetastatic niche, intravasation, extravasation, and resistance to apoptosis. Given the crucial role of hypoxia in cancer progression, hypoxic regions should be recapitulated in the TME models. Several studies have attempted to create a hypoxic environment within tumors. To precisely generate a hypoxic region, the oxygen level should be controlled and continuously monitored. For example, a device was integrated with an oxygen sensor to recapitulate the hypoxia present in breast tumor [138]. Hypoxic region was generated by incorporating a cell layer between two diffusion barriers, where an oxygen gradient is established by cellular metabolism and physical constraints. This study used an oxygen-sensitive luminophore absorbed on silica microparticles to monitor the oxygen concentrations. To precisely control and monitor the oxygen concentrations in the microfluidic device, thermoplastic polymer materials with low oxygen permeability, such as polyethylene terephthalate (PET), polycarbonate (PC), cyclic olefin copolymer (COC), or polystyrene (PS), are applied as chip materials. For example, poly(methyl methacrylate) (PMMA) was used to develop a cell culture device with controlled oxygen level. The device consists of a gas and a cell culture channel separated by a silicone membrane [139]. Optical oxygen sensors were embedded at the inlet and outlet of the culture channel, which allows for long-term monitoring of the dissolved oxygen (DO) level in the cell microenvironment. Similarly, COC was used to establish a microfluidic culture device of an HUVEC–fibroblast spheroid [140]. This study also showed that the oxygen sensor integrated within the COC device accurately detected the oxygen levels, while a PDMS device-based sensor measured higher oxygen levels, due to the high permeability to oxygen of PDMS that allows oxygen from the atmosphere to reach the channel and increased oxygen concentrations. COC and poly (methyl pentene) (PMP) were applied to develop an organ chip platform to study oxygen consumption of endothelial cells and highly active hepatocytes (HEP) [141]. In line with previous studies, HEP grown on impermeable COC devices depleted almost all oxygen within 60 min (from 15–17% to 4% of oxygen). Meanwhile, HEP grown on PEP devices induced a mildly hypoxic condition <11%) as observed in vivo.

#### 3.4.2. Measuring BBB Integrity

Although the brain is a common metastatic site, treatment of diseases at this stage is still challenging owing to numerous factors, one of which is the poor penetration of agents through the BBB, which limits their accessibility to the tumor sites. Therefore, BBB integrity and drug penetration are crucial factors in determining the treatment outcomes. TEER is a well-established method for BBB integrity measurement in vitro [142]. TEER measurements reflect the relevance of in vitro BBB models compared with in vivo studies of drug transportation and toxicity. A BBB model was developed based on the culture of brain endothelial cells on the lower side of an ECM-coated membrane with integrated TEER to monitor BBB functionality [123]. After establishing a stable BBB, tumor cells were introduced into the upper side of the membrane, and drugs were perfused into the lower channel. The penetration of anticancer drugs through the BBB was evaluated based on changes in TEER levels. 

The majority of malignant cells derived from the epithelium breach the basement membrane on the basal side and migrate to the healthy parenchyma. During this process, epithelial-mesenchymal transition plays a crucial role in cancer progression, where cells downregulate tight junction protein expression, lose their integration with other cells, and acquire migratory phenotypes [143]. Therefore, real-time monitoring of epithelial integrity provides information on epithelial dynamics during cancer progression. Several studies have integrated TEER into cell culture platforms to evaluate epithelial tightness (e.g., in the lung and gut) (Figure 4a) [132]. In addition, TEER can also be applied to determine the efficacy of anticancer drugs that cause cellular toxicity and cell detachment, leading to a reduction in the TEER value. 

**Table 2:** Organ chips integrated with biosensors for real-time monitoring of cellular behaviors and drug screening.

Organ	Tissue	Platform	Cell Types	Disorders or Diseased Models	Measurement	Types of Sensors	Year	Ref
Brain	BBB	2 culture layers separated by porous membrane	Endothelial cells, astrocytes	Normal condition	Barrier integrity	TEER	2012	[132]
	BBB	2 culture layers separated by porous membrane	Human brain endothelial cell line	Normal condition	Barrier integrity	TEER	2013	[144]
	BBB	2 culture layers separated by porous membrane	Human cerebral microvascular endothelial cells	Normal condition	Barrier integrity	TEER	2016	[145]
	BBB	2 culture layers separated by porous membrane	HiPSCs derived brain microvascular endothelial cells, rat primary astrocytes	Normal condition	Barrier integrity	TEER	2017	[146]
	Neuronal network	2 culture chambers connect by microchannels	Rat cortical neurons	Normal condition	Neuronal activity	MEA	2018	[136]
	Neuronal network	3 compartments connected by 50 microchannels	HiPSCs neurons	Epilepsy- seizure like activity	Neuronal activity	MEA	2020	[147]
Skin	Epidermis barrier with immune component	2 culture layers separated by porous membrane	Human keratinocytes, human leukemic monocyte lymphoma cell line	Normal condition	Barrier integrity	TEER	2016	[130]
	Epidermal barrier	Culture chamber with porous chamber	Human derived keratinocytes, murine fibroblast	Sodium dodecyl sulphate-induced skin irritation	Barrier integrity, extracellular acidification rate (EAR)	TEER, metal oxide sensor	2018	[148]
Lung	Bronchial epithelium	2 culture layers separated by porous membrane	Human bronchial epithelial cell line	Inflammation	Cytokines	Photonic sensor	2022	[149]
	Lung cancer cells	Printed microfluidic channel	Lung cancer cell line	Lung cancer	pH of media and cytotoxicity induced by chemotherapy	pH sensor and TEER	2020	[150]
Intestine	Gastrointestinal-microbe interface	3 culture layers separated by nano- and micro-porous membranes	Gut endothelial cells, microbe	Normal condition	Barrier integrity, oxygen	TEER, fluorescence	2016	[151]
Breast	Breast	Microwell arrays	Breast cancer cells (MDA-MB-231) and Jurkat T cells	Breast cancer	IL2 detection, T cell penetration	Optical	2021	[128]
	Breast	Matrix-based organoid cultivation integrated electrochemical sensors	Breast cancer stem cells (BCSC1)	Breast cancer	Metabolites (O_2_, glucose, lactate)	Electrochemical sensors	2022	[135]
	Breast	Culture chambers integrated with multiplexed microfluidic immunohistochemistry	Breast cancer cell lines (MCF-7, SK-BR-3, HCC70, T-47D, and MDA-MB-231) and non-tumorigenic breast cells (MCF-10A)	Breast cancer	Biomarkers (ER, PR, HER2, and Ki67)	Optical	2021	[152]
Breast + Heart	Breast + Heart	Culture chambers integrated with multiplexed microfluidic microelectrode array	Breast cancer (SK-BR-3 cell) and iPSC- derived cardiac tissues	Breast cancer; healthy/fibrotic heart tissue	Monsitoring of cell-secreted multiple biomarkers	Electrochemical immuno-aptasensors	2021	[153]

## 4. Future Consideration 

### 4.1. Current Landscape 

The OOC technology offers powerful tools that have been applied in cancer biology research and preclinical studies. For cancer modeling, cancer-on-chips allow for the recapitulation of the complexity of the TME at the organ level owing to the spatial separation of different tissues and tissue–tissue interfaces [26,154,155]. These unique architectural features enable the study of cellular behaviors within each tissue or communication among different cell types across tissues. For example, two-channel microfluidic devices with microporous separation between the upper and lower channels are among the most widely used platforms [26]. The epithelium–endothelium interface is formed by co-culturing epithelial cells on one side of the PDMS membrane and endothelial cells on the other side. These structural and functional units are useful for modeling cancer biology as most malignant cells arise from the epithelium and cross the endothelium to enter the bloodstream. To model cancer growth and invasion, cancer cells are placed within the epithelium, where they grow and migrate across the PDMS membrane and endothelium to the lower channel. To visualize cancer metastasis, the lower channel was connected to other compartments that mimicked secondary organs [102]. To date, cancer chips have focused on modeling the invasive-metastasis cascade by reconstituting tumor niches in primary organs to study cancer growth, invasion, and intravasation, whereas secondary organs provide materials for visualizing extravasation and micrometastasis [28,102]. These models not only allow for the study of cancer progression but also provide insights into how malignant cells communicate with non-malignant components in the stromal region for survival and expansion. Numerous biomolecules and signaling pathways involved in cancer progression and metastasis have been identified using these platforms, indicating that OOCs are a promising approach for investigating the underlying mechanisms of various types of cancer, including the communication of genetic and environmental factors on cancer development and prognosis [56,102]. During cancer progression, the immune system is a crucial factor that affects cancer survival and is increasingly being recognized and applied in cancer therapy [156]. Immune systems have also been integrated into cancer chips to construct physiologically relevant platforms [69]. Early studies simply introduced innate immune cells, such as macrophages, into multi-chamber co-culture systems to study the crosstalk between cancer immune cells that affect cancer invasiveness [109]. Recent studies have focused on remodeling the adaptive immune system on cancer chips to investigate how T and B cells regulate cancer development [68,156]. Another emerging approach to cancer chips is the introduction of blood cells, particularly platelets, to the TME, which reveals the significant impact of these cells on cancer progression by promoting extravasation [69,90,157]. Taken together, OOC technology offers a powerful approach to cancer biology research, which has been extensively applied to recapitulate the complexity of the TME and explore the underlying mechanisms of cancer progression and therapeutic resistance. 

### 4.2. Arising Approaches 

#### 4.2.1. Synergistic Approach of Cancer-on-Chips and Cancer Organoids

Recently, organoids and organs-on-chips have been developed because of their ability to mimic the human body anatomically and physiologically. An organoid is a self-organized 3D tissue that is typically derived from stem cells (pluripotent or tissue-resident) or progenitor cells and is directed to differentiate into multiple tissues (e.g., gut organoid, kidney organoid, brain organoid), which mimic the development process or morphogenesis and form a variety of miniature organs that recapitulate the structure and function of their in vivo counterparts [158]. During development, organoids mainly rely on biochemical cues (e.g., growth factors) that direct the differentiation of stem cells into distinct lineages. Organ-on-chip is a bioengineering approach that utilizes the unique properties of microfluidic channels to reconstitute the architecture, structure, and function of organs. Organ chips are usually compartmentalized into distinct channels representing different tissues and tissue–tissue interfaces. Because of the different approaches, organoids and organ-on-chip have several properties that distinguish them from each other. For example, organoids mainly rely on intrinsic programming signaling, which results in highly variable cell populations, structures, and functions. The structure and geometry of the organ chips were designed precisely. In addition, organoids primarily contain one tissue type, such as epithelium, but do not involve other types of tissues within the same organs (e.g., cells of the blood vessels and resident-immune cells). An organ-on-chip consists of several tissues (epithelium, blood vessels, and immune cells) within a single device. Another crucial advantage of organ chips is that multiple biophysical components, especially flow, can be integrated to precisely control cellular behavior. In general, each approach has some disadvantages that can be compensated for by combining it with another approach to create superior platforms [159,160]. Organoids are self-organization structures developed by the intrinsic programming of human stem cells into distinct organs with an architecture resembling that of human organs. They are static cell culture platforms that lack precise control of flow input and output, nutrient supply, and biochemical as well as the biophysical microenvironment. They also lack tissue and organ interactions, all of which can be compensated by growing organoids onto the OOCs platform [159]. In cancer research, organ chips have been widely applied to model disease progression (invasion-metastasis cascade) because of the presence of multiple tissue types that recapitulate the complex TME involving the interaction of tumor cell–ECM–blood vessel–immune cells that facilitate cancer development. Cancer organoids can be derived from patient cancer stem cells, which differentiate into heterogeneous tumor cells resembling tumors in vivo. By embedding these cancer organoids into microfluidic devices, these tumor “organ” platforms enable crosstalk between the tumors and surrounding stroma, vasculature, and immune organs. The integration of organoids with organ-on-chip technology is a convergence of biotechnology and bioengineering, providing a promising approach to understanding cancer cell biology and anticancer drug development.

#### 4.2.2. Vascularized-Human-on-a-Chip in Cancer Research

Extensive efforts have been made to combine multiple systems into a single “body-on-a-chip” to model diseases and drug metabolism [161,162]. Each organ of the human body is not isolated from others but is integrated into a precisely controlled system. Any change or disturbance in a single organ affects the whole body. Failure of any organ triggers injury to other organs and causes long-term irreversible damage. All organs in the human body are nourished and interconnected via the cardiovascular system with the heart at the center and a condensed vascular network carrying its branches to every tissue to transport oxygen, CO_2_, nutrients, and metabolites. Furthermore, the vascular system transports cancer cells from their original site to distant organs throughout the body. Therefore, vasculogenesis chips integrated with multiple organs are powerful tools for studying cancer biology and for developing anticancer therapeutics [161]. Those models are of importance to monitor the dynamic of the tumor. For example, after injecting cancer cells into a primary organ, their proliferation, invasive, and migration rate to other organs can be evaluated and determine the key factors affecting the metastasis rate. The anticancer drugs do not only induce cytotoxicity on cancerous cells, but also can cause the adverse effect on other organs (e.g., heart, liver, kidney), which is a crucial aspect to consider when deciding the dosages of drugs applied for cancer patients [163]. To precisely recapitulate the drugs’ responses in patients, multi-organ chips are of great importance as they contain not only tumor but also healthy organs that help to evaluate the anti-cancer efficacy, toxicity on healthy tissues, as well as drug kinetics at the same time. These platforms can be generated by combining different single-organ chips that are connected by microfluidic channels lined by endothelial cells. Considerable efforts have been made to achieve this, including both vascularized single-organ chips and multiorgan chips with vessel channels (Table 3). Single-vascularized COCs, including endothelialized channels and vasculogenesis/angiogenesis chips, have been developed to investigate the mechanisms underlying intravasation, extravasation, and micrometastasis at the primary and distant organ separately. These single-organ chips were integrated to model the invasion-metastasis cascade and identify the fundamental mechanistic basis of this biological process. For example, a lung-on-a-chip and BBB-brain parenchyma-on-a-chip were connected to study the invasion of lung cancer cells through the BBB into the brain parenchyma [102]. In this device, both the lung and brain included the blood vessel channel lined by endothelial cells. In addition to simulating the metastasis cascade, integrated COCs are promising approaches for enhancing our understanding of drug metabolism. For instance, a linkage system consisting of the intestine, liver, and GBMs has been developed to evaluate therapeutic efficacy in GBMs [164]. Orally administered drugs pass through the intestine into the liver compartment, are metabolized into active forms, and finally reach GBMs. Although robust attempts have been made to construct an in vivo-like human-body-on-a-chip, these multiplex systems face several challenges, including scaling, vascularization, and nerve innervation. The blood vessels in vivo not only connect organs but the endothelial cells play a crucial role in transporting nutrients and metabolites; therefore, these cells can be considered to be located in the microchannels that connect different organ chambers within a device. It is necessary to scale a macroscale human organ into microscale OOCs and then scale multiple single OOCs into multiplex OOCs. The development of human-on-a-chip will help understand cancer systematically and provide valuable platforms for preclinical drug evaluation. 

### 4.3. Biological Challenges

Although it has several advantages over static cell culture platforms and has been increasingly used in cancer modeling and therapeutic development, cancer chip technology still faces several challenges that need to be solved before it can be widely applied in cancer research and clinics. The first challenge is to precisely recapitulate the complexity of the TME of its in vivo counterpart. The TME is composed of cancer cells and non-cancerous components, including healthy parenchyma cells, stromal cells, ECM, interstitial fluid, blood/lymphatic vessels with circulating blood cells, and the immune system, including both resident cells and circulating cells. Current OOCs are commonly constructed based on only three to five factors in addition to flow, which is an intrinsic factor of microfluidic devices [168,169]. The simplicity of these organ chips has benefits as the complex systems are dissected into simpler parts that allow for investigation of the influence of another component in the TME on cancer progression. However, these systems might not faithfully reflect the cellular behaviors occurring in vivo. For example, within tumors, immune cells are recruited and activated into cancer-associated phenotypes that further support cancer cell survival and invasiveness [170]. Therefore, in a system lacking immune components, the development of cancer cells might be less severe in vivo, which can lead to improper responses of these cells to anticancer drugs. Another challenge of the COC model is the introduction of a cancer-specific ECM into devices. Current cancer chips mainly use commercialized matrices, such as collagen I, laminin, and fibronectin, to model the TME, although these materials contain only a single structural protein that poorly reflects the heterogeneity of the tumor ECM [171]. As the roles of the ECM in cancer progression are increasingly recognized, organ-, tissue-, and disease-specific materials should be applied for more precise recapitulation of the TME.

### 4.4. Technical Challenges

Although the number of published papers using OOCs for cancer studies and drug screening has increased extensively over the last decade, the applications of cancer chips in cancer research and drug development are still limited by several technical challenges. A basic problem that must be overcome is the material used for the fabrication. Most organ chips are made of PDMS, which has several advantages for use as a cell culture device: biocompatibility, optical transparency, gas permeability, flexibility, and low cost. However, the use of PDMS has raised concerns, mostly because of its ability to absorb hydrophobic molecules [172,173]. This might have led to the absorbance of hydrophobic drugs into the PDMS, resulting in a reduction in drug concentration and inaccurate evaluation of cellular responses. To overcome this problem, other materials, such as PS and PMMA, can be considered alternatives for chip fabrication. Furthermore, to expand OOC applications and increase their acceptance in research, it is necessary to simplify chip preparation through high-throughput fabrication. Another problem of cancer chips that must be addressed is the availability of cell sources. Most types of cells used in these platforms are derived from immortalized cell lines, which have been genetically modified and may affect cellular behaviors and responses to therapy. Another concern is that cancer cells are highly heterogeneous within tumors or among individuals. Therefore, cell lines may have distinct genetic backgrounds from patient cells and do not provide precise information on cellular signaling and therapeutic responses. Patient-derived iPSCs that undergo genetic engineering may serve as potential cell sources to model patient-specific TME and provide more accurate therapeutic responses that help select the appropriate treatment for each individual [174,175]. As the complexity of cancer chips continues to increase to model the physiologically relevant TME and connect multiple organs for drug monitoring, these systems face other challenges regarding the maintenance of various cell types derived from different tissues and organs on a single platform. While each cell type requires multiple cytokines to maintain viability and to properly function, growth factors supporting one cell type can inhibit the activity of others; growing several types of cells on a single integrated device is a challenge that must be overcome to achieve the goal of modeling multiorgan or body-on-a-chip.

## 5. Conclusions and Outlook

The evolution of in vitro cell culture platforms has led to the emergence of OOC. This microfluidic-based approach attempts to recapitulate the complexity of human anatomy and physiology by focusing on the functional unit of each organ and fluid dynamics. Compared to other in vitro models, OOC is a more biologically and physiologically relevant model for reconstituting the tissue–tissue interface and organ–organ connection via microfluidic chambers and channels, which cannot be achieved using static cell culture platforms [176]. Microfluidic cell culture devices have been used to culture cancer cells for almost 20 years [19,20]. The first COC devices cultured cancer cells in “organ chambers” and were used to evaluate cancer cell viability and proliferation in long-term culture [19,20]. In recent decades, COCs have focused on modeling metastasis and the cancer niche on a chip [28], thereby applying these platforms for anticancer drug testing. COCs have successfully recapitulated the metastatic cascade and cancer development through multiple stages [28], which is also in line with clinical observations. An example of this application is breast cancer-on-a-chip, in which all stages of the metastasis cascade have been modeled, ranging from ductal in situ carcinoma [44], stromal invasion, intravasation [177], extravasation [50], and metastasis [56]. Unlike other types of cancer, GBM rarely metastasizes to secondary organs; therefore, for brain cancer on-chip or GBM chip, the focus has been the modeling of a tumor niche instead of the metastasis cascade. Three dominant types of niches found in GBM, including perivascular, invasive, and hypoxic niches, have been remodeled on a chip, which successfully mimics pathogenesis hallmarks, including hyperangiogenesis [77], cancer cell invasion [81], and pseudopalisading necrosis [83], respectively. Furthermore, COCs do not simply reconstitute the histological and pathological characteristics of each cancer type but also identify the mechanisms of these diseases. In addition to cancer biology studies, COCs have been designed as platforms to examine anticancer drug responses. Failure in clinical trials has led to questions regarding the relevance of animal models in predicting human drug responses [19]. This emphasizes the importance of in vitro human cancer models for preclinical drug testing. In this regard, OOCs have several advantages over static cell culture models because of their ability to circulate drugs and metabolites between tissues and organs. In the last decade, anticancer drugs, particularly chemotherapy, have been applied to TME COCs and have shown more physiologically relevant responses. Patient-specific samples were also embedded into these devices, and the observed responses to anticancer drugs were compatible with clinical data [84] (e.g., genetic background, drug resistance, and prognosis). These results emphasize the importance of a physiologically relevant TME, including blood vessels, lymphatic vessels, ECM, and interstitial flow, as preclinical models [162]. Integrating a circulation system with multiple organs, including those where drugs are metabolized (liver and kidney), is essential for constructing a platform for testing toxicity and adaptation to cancer therapy. Furthermore, biosensors are critical components in advansced COCs that should be intensively considered when designing an integrated COC with biosensors on the chip. This would contribute to real-time monitoring of oxygen, metabolites, stress indicators (ROS), and electrophysiological activities to precisely construct a TME and evaluate cancer progression as well as responses to anticancer therapy. We believe that advanced cancer-on-chip technology that recapitulates TMEs and integrates biosensors within the chip or MAP will significantly impact life sciences and high-throughput drug screening.

## Figures and Tables

**Table 1:** High throughput platforms for anticancer drug screening.

Organ	Type of Cancer	Platform	Structure	Cell Types	Therapy	Testing Effect	Year	Ref
Breast	Breast	Microfluidic U-shape arrays	Tumor spheroids	Human breast cancer cells, MCF7	Chemotherapy	Dynamic transport behavior Cytotoxicity	2008	[43]
	Breast	U-shaped arrays connecting with 2 parallel channels	Vessel-ECM-tumor	HUVECs, breast cancer cells (BT549, T47D)	Chemotherapy	Dynamic transport behavior Cytotoxicity	2018	[45]
	Breast	Microfluidic arrays	Tumor-healthy cells	Breast cancer cells (MDA-MB–231) and normal breast cells (HMEpiC)	Chemotherapy	Inhibition of migration	2016	[125]
	Breast	Microfluidic arrays	Tumor/healthy cell droplets	Breast cancer cells (MDA-MB-231) or normal breast cells (MCF-10A)	Chemotherapy	Cytotoxicity	2021	[126]
	Breast	Concentration gradient generator	2D tumor	Breast cancer cells (MCF7)	Chemotherapy	Cytotoxicity	2011	[127]
	Breast	Microfluidic arrays	Tumor spheroids	MCF-7 cells	Chemotherapy	Cytotoxicity	2022	[119]
	Breast	Microwell arrays	Tumor spheroids	Breast cancer cells (MDA-MB-231) and Jurkat T cells	Immunotherapy	Interleukin (IL) 12 detection, T cell penetration	2021	[128]
Brain	Lung, GBM	24 units of 2-channel device	Tumor-BBB	Brain microvascular endothelial cells (hCMEC/D3), lung cancer cells (PC-9) and glioma cells (U251)	Chemotherapy	Drug permeability, Cytotoxicity on tumor and BBB	2022	[123]
	GBM	Concentration gradient generator	Tumor spheroids	Glioblastoma cells (U87)	Chemotherapy	Cytotoxicity	2016	[118]
	GBM	Microfluidic arrays	Tumor spheroids	Glioblastoma cell (U251)	Chemotherapy	Cytotoxicity	2015	[129]

**Table 3:** Multi-organ microfluidic chips for cancer modeling and drug screening.

Organs	Types of Cancer	Advantages of Multi-Organ Platform	Year	Ref
Liver + Brain	Brain	Evaluation of drug metabolism in the liver and BBB penetration	2021	[123]
Colon + Intestine + Liver	Colon	Demonstration of metastatic phenotype in vitro Evaluation of effect of chemotherapy on tumor migration	2016	[165]
Small intestine + Liver + Lung	Colon, liver, lung	Pharmacokinetic model mimicked internal circulation of intestine, liver, and lung with in vivo like volume and flow ratio	2015	[166]
Heart + Muscle + Brain + Liver	Liver	Evaluation of multi-organ drug toxicity	2016	[167]
Breast + Heart	Breast	Evaluation of chemotherapy-induced cardiocytoxicity	2021	[153]

## Data Availability

Not applicable.
